# Jejunal inflammatory cytokines, barrier proteins and microbiome-metabolome responses to early supplementary feeding of Bamei suckling piglets

**DOI:** 10.1186/s12866-020-01847-y

**Published:** 2020-06-17

**Authors:** Jipeng Jin, Jianlei Jia, Liping Zhang, Qian Chen, Xiaoyan Zhang, Weibo Sun, Cunming Ma, Fafang Xu, Shoujun Zhan, Limin Ma, Guihua Zhou, Qiaoxi Chen

**Affiliations:** 1 College of Animal Science and Technology, Gansu Agricultural University, Lanzhou, 730070 China; 2 Key of laboratory of Plateau Ecology and Agriculture, Qinghai University, Xining, 810016 China; 3 College of agriculture and Animal Husbandry, Qinghai University, Xining, 810016 China; 4 Qinghai Province Huzhu County Bamei Pig Seed Breeding Farm, Huzhu, 810500 China; 5 Pingliang Mechanical and Electrical Engineering School, Jingning, 743417 China

**Keywords:** Bamei suckling piglets, Jejunal microbiota, Metabolic profiles, Jejunal barrier

## Abstract

**Background:**

Dietary intervention has been reported to improve intestinal health. The intestinal microbiota of newborn animals plays a fundamental role in the development of intestinal function and the innate immune system. However, little is currently known about dietary interventions in the gut microbiota and barrier function of livestock, especially suckling Bamei piglets. To this end, we studied the effect of early dietary supplementation on intestinal bacterial communities and intestinal barrier function in piglets.

**Results:**

10 purebred Bamei sows were randomly allocated into two groups. In group one, the piglets received a supplementary milk replacer on day 7 of age, whereas the other control group was allowed sow’s milk alone. At 21 days, 18 and 17, respectively, piglets in each group of average weight were randomly selected and sacrificed. Tissue and digesta samples were collected from the jejunum to evaluate differences in the microbiome-metabolome and the mRNA expression of inflammatory cytokines (TLR4, TNFα and IL-8) and barrier proteins (ZO-1, Occludin and Claudin-1). Sequencing of 16S rRNA revealed that ES improved the gut microbiome composition of Bamei suckling piglets. The relative abundances of some bacterial species such as *Lactobacillales*, *Romboutsia*, *Actinobacillus*, *Bacteroides* were significantly reduced in the ES group. Metabolomics analysis indicated that 23 compounds were enriched and 35 compounds decreased in the ES group. And correlation analysis demonstrated that some gut bacterial genera were highly correlated with altered gut microbiota-related metabolites. Meanwhile, ES of Bamei suckling piglets altered the gene expression of inflammatory cytokine and barrier protein in the jejunum.

**Conclusions:**

In summary, these results provide important insights on the relationships between jejunal microbiota and related metabolites, and jejunal barrier function during the early life of Bamei suckling piglets.

## Background

As is well known, the gastrointestinal tract of humans and animals is a highly diverse ecosystem [1]. The mammalian intestine is colonized by many thousands of microbiota strains with the total number of microbial cells exceeding other parts. The gut microbiota provides various benefits to the host, including nutrient absorption, metabolism, developing the immune defense systems, intestinal epithelial differentiation, and intestinal mucosal barrier maintenance, which all play a key role in human health and disease [2–6].

The gut microbiome has long been known to have fundamentally important roles in the health and the well-being of its host. Therefore, it is very important to establish and maintain the beneficial gut microbiota in the pig’s early life, because those early gut colonizers are very important in establishing the permanent microbial community structures for impacting, the health and growth performance of rapidly growing piglets. The microorganisms present in breast milk have a significant role in the newborn’s developing immune system [7]. Also other factors affect intestinal microbial diversity, such as, age, sex, and environment [8, 9]. However, dietary interventions can improve health via increasing bacterial abundance [10–13]. Yi et al. reported that dietary supplementation with a 1% amino acid blend improved intestinal functions and reduced the incidence of diarrhea in piglets [14]. Geng et al. assessed the effect of fecal microbiota transplantation (FMT) on intestinal homeostasis through early intestinal microbial intervention resulting in a subsequent lipopolysaccharide attack in newborn piglets. It was observed that FMT could regulate tryptophan metabolism by the newborn piglets intestinal microflora, potentially contributing intestinal barrier maintenance [15]. Yang et al. demonstrated that early supplementation with Alfalfa stimulated changes to the ruminal microflora both before and after weaning [16]. Using high-throughput sequencing of the 16S rRNA gene, Wang et al. reported that the colostrum microbiota is significantly different from the microbial population observed in the feces of both calf and cattle. The calf feces microbial population may in fact be derived from the birth canal at the time of parturition [17]. In a recently published study, de Goffau et al. overturned the currently held notion that the placenta contains its own microbiota [18] through a detailed analysis of large sample numbers. In fact, it was determined that the majority of bacterial species observed in the placenta are likely derived from contamination, because presence is not established in the placenta before fetal birth [19]. Related research demonstrates that small intestine villi height increased up to d 7 and then declined from d 7 to d 21 [20]. The mRNA relative abundance of ZO-1 in the small intestine mucosa was highest on d 7 [21]. This implies that after the birth of Bamei piglets, the diet has a great influence on intestinal microbiome development. However, little scientific information is known regarding the relationships and responses between the jejunal inflammatory cytokines, barrier proteins, and microbiome-metabolome when feeding supplements to suckling Bamei piglets.

In this study, a combination of methods including. qRT-PCR, 16S rRNA gene sequencing, and liquid chromatography-mass spectrometry (LC-MS) metabolomics were used to analyze the responses to early dietary supplementation to suckling Bamei piglets on their jejunal immunity and barrier function, microbiome, and metabolite profiles. It was observed that early supplementation significantly altered the gene expression of inflammatory cytokines and barrier proteins in the jejunum of Bamei suckling piglets. Furthermore, early dietary supplementation significantly improved the jejunal microbiome composition, which resulted in a demonstratable improvement of the metabolites produced by the microbiota.

## Results

### Weight gain, diarrhea incidence, and mRNA expression of inflammatory cytokines and barrier proteins in suckling Bamei pigs

**Fig. 1:**
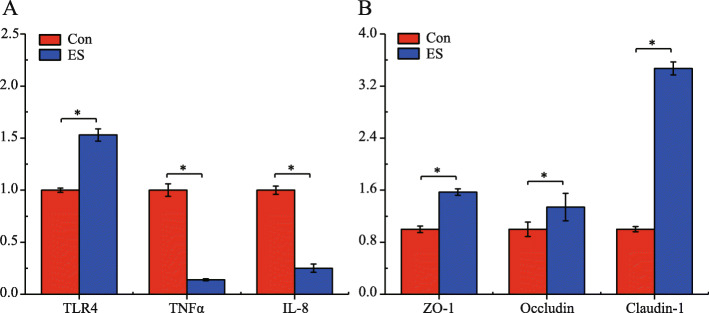
Inflammatory cytokines (**a**) and tight junction proteins (**b**) gene expression in the jejunum of Bamei suckling piglets without feeding or early supplementary feeding. Error bars show standard deviation. *p < 0.05

As can be summarized from Table 1, piglets fed the supplement (ES) demonstrated significantly increased (*p* < 0.05) final body weight and ADG compared with piglets in the Con group, while diarrhea incidence was significantly decreased for piglets fed ES compared with Con piglets (*p* < 0.05). Early dietary supplementation had a substantial effect on the expression of inflammatory cytokines and tight junction protein genes in the jejunum (Fig. 1). In the ES group, TLR4, ZO-1, Occludin and Claudin-1 mRNA levels were higher compared to the Con group (*p* < 0.05). For Bamei piglets fed ES, jejunum TNFα and IL-8 mRNA concentration were significantly lower compared to Bamei piglets in the Con group (*p* < 0.05).

**Table 1:** Weight gain and diarrhea incidence of Bamei Suckling piglets fed with or without dietary ES

Variable	Con	ES	*p*-value
Initial body weight, kg	0.88 ± 0.13	0.89 ± 0.12	0.661
Final body weight, kg	4.66 ± 0.90	5.01 ± 0.71	0.025
ADG, g/d	0.18 ± 0.04	0.20 ± 0.03	0.031
Diarrhea incidence, %	8.83 ± 0.40	5.34 ± 0.37	0.001<

### ES induced changes to the jejunum microbiome of Bamei suckling piglets

**Fig. 2:**
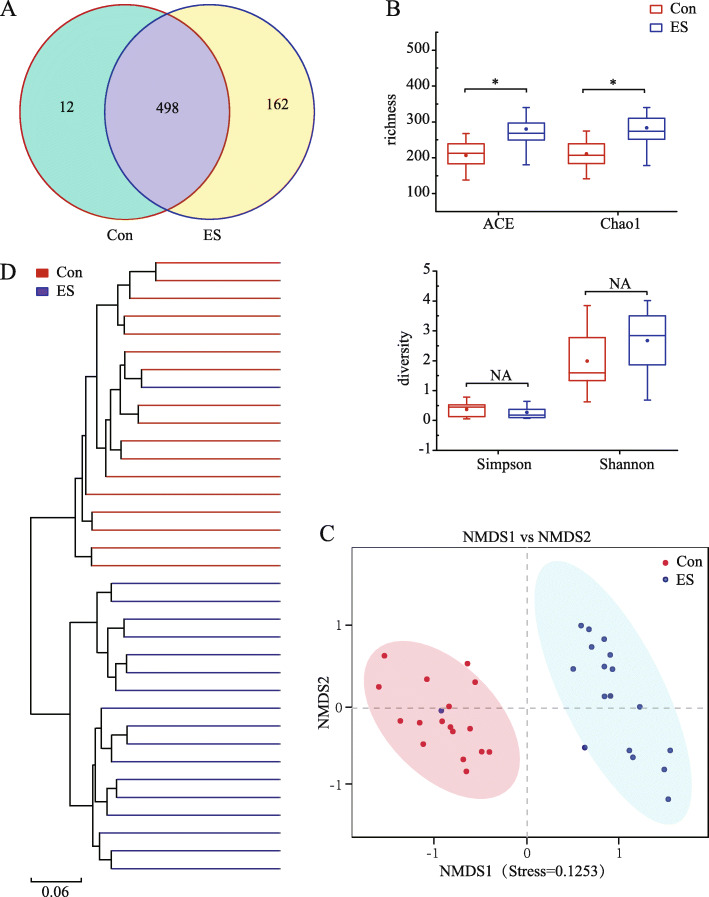
Differences in bacterial community diversity, richness, and structures in the jejunum of Bamei suckling piglets without feeding or early supplementary feeding. **a** Venn diagram of shared OTUs. **b** Community diversity and richness. **c** Non-MetricMulti-Dimensional Scaling (NMDS) plot, (**d**) Unweighted Pair-group Method with Arithmetic Mean (UPGMA) tree. Con: without feeding; ES: early supplementary feeding. *p < 0.05

An average of 61,438 clean tags per sample were obtained from the 35 jejunal content samples analyzed. A total of 672 OTUs based on a 97% sequence similarity were identified from these sequences. Of these 672 OTUs, 498 OTUs were common among both groups (Fig. 2a). These 498 OTUs mapped to 20 phyla, 48 classes, 106 orders, 182 families, 350 genera, and 377 species. The alpha diversity was estimated through the diversity index (Shannon and Simpson) and richness estimate (Chao1 and Ace). As can be seen in Fig. 2b, the richness estimate (ACE and Chao1) increased significantly for ES group compared to the Con group (*p* < 0.05), whereas the diversity indices (Simpson and Shannon) were similar among both groups (*p* > 0.05). The NMDS plot (Stress = 0.125 < 0.2), which is used to illustrate the dissimilarity of the microbial community, revealed distinct structures between the Con and ES groups (Fig. 2c). Similarly, the jackknifed beta diversity and hierarchical clustering analysis via the UPGMA demonstrated that different groups were clustered into their own groups (Fig. 2d). These results suggest the gut microbiota composition in suckling Bamei pigs was being altered by early dietary supplementation.

**Fig. 3:**
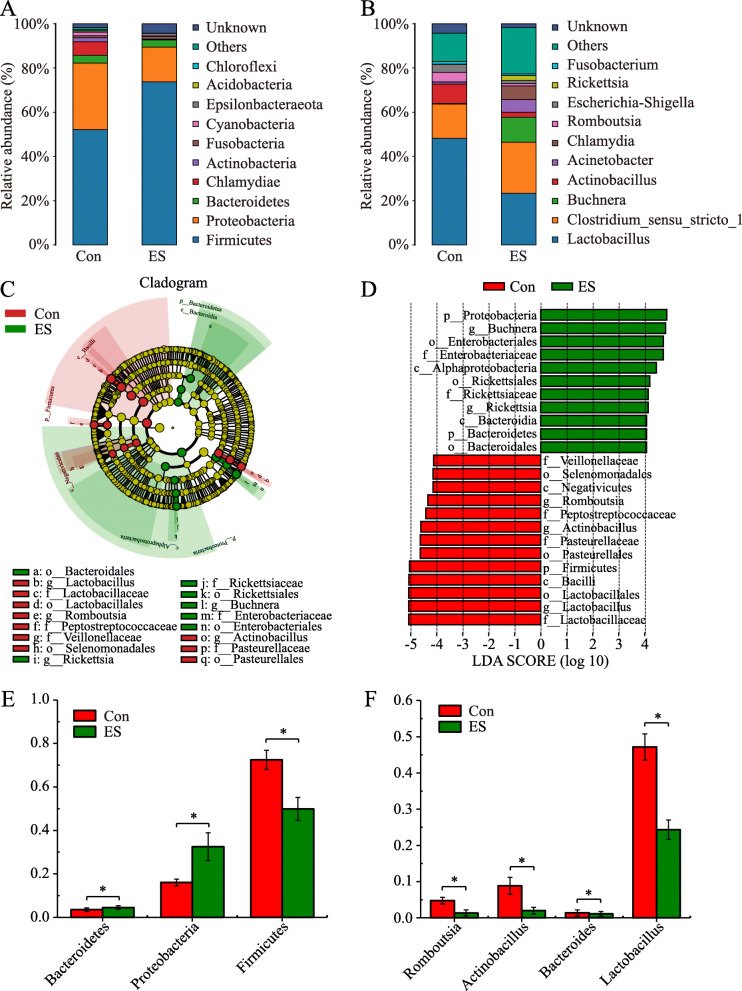
Changes of microbial composition in the jejunum of Bamei suckling piglets without feeding or early supplementary feeding. Microbial composition at the phylum level (**a**) and genus level (**b**) each bar represented the average relative abundance of each bacterial taxon within a group. Cladogram (**c**) and LDA value distribution histogram (**d**). Kruskal-Wallis rank sum test analysis histogram (**d**). The bacterial phyla (**e**) and genera (**f**). Con: without feeding; ES: early supplementary feeding. *p < 0.05

Here, a total of 20 phylum and 350 genera were identified within the jejunum microbiome. Figures 3a, b report the top 10 most abundant microbes in the piglet’s jejunum intestinal contents for both groups. *Firmicutes* and *Proteobacteria* were observed as the predominant phyla in the jejunal microbiota, followed by *Bacteroidetes*, *Chlamydiae*, and *Actinobacteria*. The relative jejunal microbiota abundances at the genus level are presented in Fig. 3b. The results demonstrate that *Lactobacillus*, *Clostridium_sensu_stricto_1*, *Buchnera*, *Actinobacillus*, and *Acinetobacter* were the predominant genera. Linear discriminant analysis (LDA) effect size taxonomic cladogram (Fig. 3c) and LDA value distribution histogram (Fig. 3d) were used to determine the jejunal microbiota structure and the predominant bacteria present with the greatest taxa differences between the two groups displayed (i.e. LDA score > 4). The relative abundances of *Proteobacteria*, *Buchnera*, *Enterobacteriales*, *Enterobacteriaceae*, *Alphaproteobacteria*, *Rickettsiales*, *Rickettsiaceae*, *Rickettsia*, *Bacteroidia*, *Bacteroidetes*, and *Bacteroidales* were significantly higher for piglets fed ES, while the relative abundances of *Veillonellaceae*, *Selenomonadales*, *Negativicutes*, *Romboutsia*, *Peptostreptococcaceae*, *Actinobacillus*, *Pasteurellaceae*, *Pasteurellales*, *Firmicutes*, *Bacilli*, *Lactobacillales*, *Lactobacillus*, and *Lactobacillaceae* were reduced compared to piglet’s fed control. The data from the Kruskal-Wallis rank sum test analysis histogram of bacterial phyla and genera data is presented in Figs. 3e, f. The relative abundances of *Bacteroidetes*, *Proteobacteria* and *Firmicutes* accounted for more than 1% of the total microbiome. For piglet’s fed ES the relative abundance of *Bacteroidetes* and *Proteobacteria* were significantly decreased (*p* < 0.05), and *Firmicutes* was significantly lower compared with piglets fed Con (*p* < 0.05). Seven (7) genera demonstrated relative abundances of more than 1%, whereas piglet’s fed ES demonstrated lower (*p* < 0.05) *Romboutsia*, *Actinobacillus*, *Bacteroides* and *Lactobacillus* were lower than piglet’s fed Con.

**Fig. 4:**
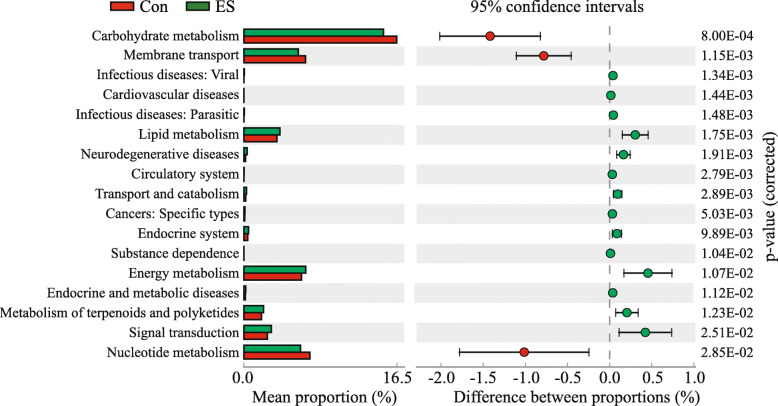
KEGG metabolic pathway difference analysis. The figure shows the abundance ratio of the different functions in the Con and ES groups. The middle shows the difference in functional abundance in the 95% confidence interval, and the right most value is the *p*-value (corrected). Con: without feeding; ES: early supplementary feeding

In order to evaluate the functional capacity of the jejunal bacterial communities for both piglet groups, PICRUSt was used to further analyze the KEGG pathway compositions. The second level KEGG pathway analysis showed that Lipid metabolism, Energy metabolism, Metabolism of terpenoids and polyketides, Infectious diseases: Viral, Cardiovascular diseases, Infectious diseases: Parasitic, Neurodegenerative diseases, Circulatory system, Transport and catabolism, Cancers: Specific types, Endocrine system, Substance dependence, Endocrine and metabolic diseases, and Signal transduction were enriched (*p* < 0.05), while Carbohydrate metabolism, Nucleotide metabolism and Membrane transport were decreased (*p* < 0.05) in the ES group (Fig. 4).

### Metabolites and metabolic pathways within the jejunum

**Fig. 5:**
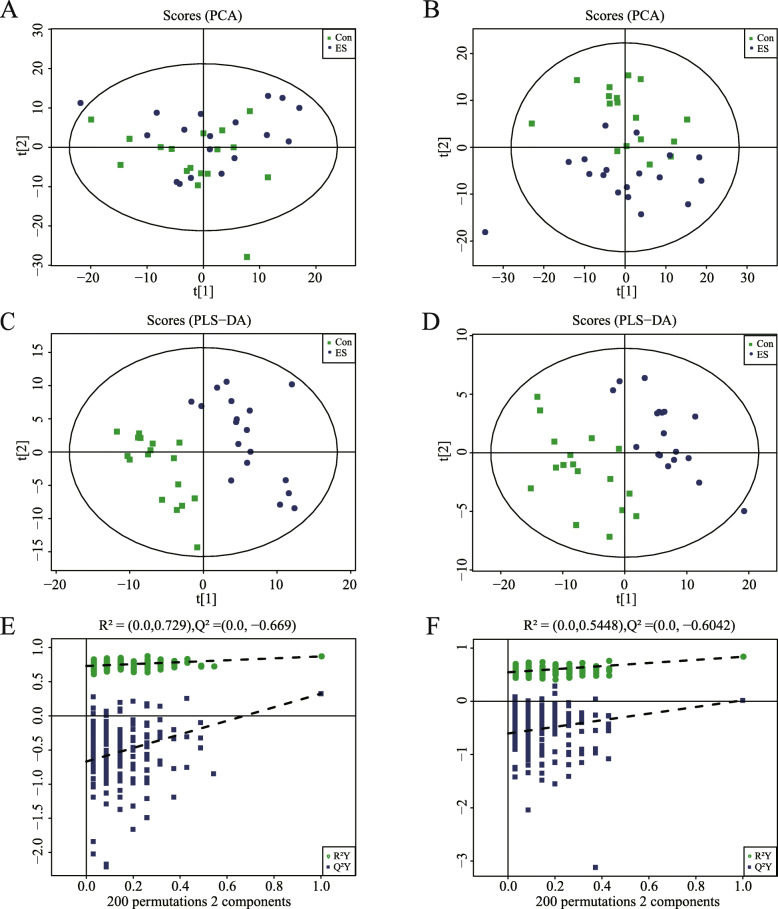
Plot of PCA (**a**) and OPLS-DA (**c**), R2X 0.184, R2Y 0.869, Q2 0.471) score, and OPLS-DA permutation (**e**) in the positive model of the Bamei suckling piglets jejunal samples corresponding to the comparison between Con and ES group. Plot of PCA (**b**) and OPLS-DA (**d**), R2X 0.182, R2Y 0.836, Q2 0.321) score, and OPLS-DA permutation (**f**) in the negative model of the Bamei suckling piglets jejunal samples corresponding to the comparison between Con and ES group. Con: without feeding; ES: early supplementary feeding

To further explore early dietary supplementation influence on jejunal microbiota, the jejunum metabolite concentrations for both groups were analyzed. A total of 283 metabolites were found. These metabolites, including amino acids, carbohydrates, organic acids, lipids, nucleotides, and others, are involved in multiple jejunum biochemical processes for the Bamei piglets. The PCA score plots from were derived from the LC-TOF/MS metabolic profiles of jejunal contents showed separation between piglets fed ES and Con (Fig. 5a, b). As shown in the OPLS-DA score plot (Fig. 5c, d), piglets fed ES compared to groups and piglets fed Con could be separated into distinct clusters according to their metabolic differences (OPLS-DA models +: R2Y = 0.869 and Q2 = 0.471; −: R2Y = 0.836 and Q2 = 0.321). In addition, the permutation test for OPLS-DA demonstrated the Q2 regression line had a negative intercept. Additionally, all R2 and Q2 values on the left were lower than the original points on the right (OPLS-DA validate models +: R2Y = 0.729 and Q2 = − 0.669; −: R2Y = 0.5448 and Q2 = − 0.6042) (Fig. 5e, f), demonstrating that the OPLS-DA model in the present study is valid.

**Fig. 6:**
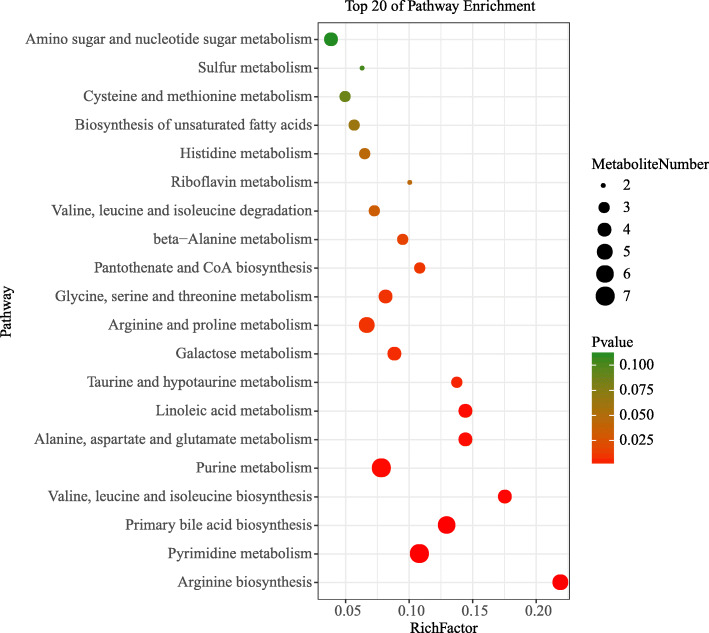
Metabolic pathway enrichment analysis. Overview of metabolites that were enriched in the jejunum of Bamei suckling piglets without feeding or early supplementary feeding

To identify compound differences between the two treatment groups, the parameters of VIP > 1 and *p* < 0.05 were used as a criterion. In the jejunum, 23 compounds (L-Citrulline, Betaine, 1,2-dioleoyl-sn-glycero-3-phosphatidylcholine, 5-Methylcytosine, Cytosine, Glycitein, Daidzein, N-Oleoylethanolamine, L-Histidine, Acetylcarnitine, 1-Myristoyl-sn-glycero-3-phosphocholine, 1-Oleoyl-L-.alpha.-lysophosphatidic acid, 2′-Deoxyinosine, 1-Palmitoyl-sn-glycero-3-phosphocholine, Thioetheramide-PC, PC (16:0/16:0), Linoleoyl ethanolamide, Thymine, Arachidonic Acid (peroxide free), 2′-Deoxyuridine, Genistein, Chenodeoxycholate, 4-Androsten-17.beta.-ol-3-one glucosiduronate) were increased and 35 compounds (Guanosine, Pro-Glu, 2-Hydroxyadenine, N-Acetylmannosamine, N-Acetylneuraminic acid, Pro-Ala, Uridine, N-Acetyl-D-glucosamine, L-Arginine, Pro-Thr, Pro-Phe, MG (18:2(9Z,12Z)/0:0/0:0)[rac], Allopurinol riboside, Ile-Pro, Asp-Leu, Thr-Ala, Riboflavin, Cholic acid, Hypoxanthine, S-Methyl-5′-thioadenosine, Trimethylamine N-oxide, Adenine, 3-Methoxy-4-Hydroxyphenylglycol Sulfate, Muramic acid, all cis-(6,9,12)-Linolenic acid, Lumichrome, Alpha-D-Glucose, D-Mannose, Pantothenate, L-Asparagine, D-Lyxose, L-Threonine, L-Aspartate, Inosine) were decreased for piglets fed ES compared to piglets fed Con (Table S1). Further metabolic pathway enrichment analysis demonstrated that piglets fed ES significantly altered their arginine biosynthesis, pyrimidine metabolism, primary bile acid biosynthesis, valine, leucine and isoleucine biosynthesis, alanine, aspartate and glutamate metabolism, linoleic acid metabolism, taurine and hypotaurine metabolism, pantothenate and CoA biosynthesis, and riboflavin metabolism (*p* < 0.05, rich factor > 0.1, Fig. 6) compared to piglets fed Con.

### Correlation analysis

**Fig. 7:**
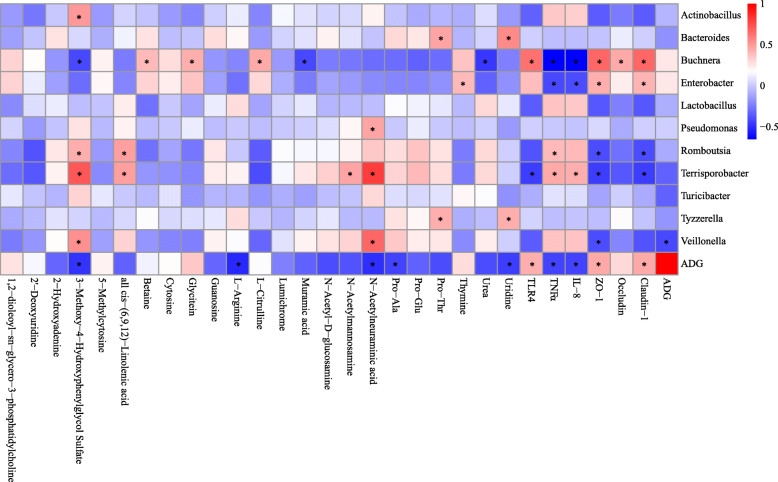
Correlation analysis in the jejunum of Bamei suckling piglets without feeding or early supplementary feeding. Red represents a positive correlation, while blue represents a negative correlation. *Significant correlation between the ES and Con groups (*p* < 0.05)

Metabolites with VIP > 1 (*p* < 0.01) and genera with significantly different abundances (*p* < 0.05) between piglets fed ES and Con were used for the Pearson’s correlation coefficient analysis (*r* > 0.4 or < − 0.4, *p* < 0.05). As is shown in Fig. 7, the relative abundance of 3-Methoxy-4-Hydroxyphenylglycol Sulfate was positively correlated with *Actinobacillus*, *Romboutsia*, *Terrisporobacter*, and *Veillonella*, while negatively correlated with *Buchnera*. The relative abundance of N-Acetylneuraminic acid showed positive correlations with *Terrisporobacter*, *Pseudomonas*, *Terrisporobacter*, and *Veillonella*. It was also observed that Pro-Thr and Uridine levels were positively correlated with *Bacteroides* and *Tyzzerella*. Furthermore, cis-(6,9,12)-Linolenic acid was positively correlated with *Romboutsia* and *Terrisporobacter*. Thymine was positively correlated with *Enterobacter*. *Buchnera* was positively correlated with Betaine, Glycitein, and L-Citrulline, and negatively correlated with Muramic acid and Urea. Levels of TLR4 were positively correlated with the relative abundances of *Buchnera*, but was negatively correlated with the relative abundance of *Terrisporobacter*. Conversely, TNFα was positively correlated with the relative abundances of *Romboutsia* and *Terrisporobacter*, and was negatively correlated with the relative abundance of *Buchnera* and *Enterobacter*. Similarly, IL-8 was positively correlated with the relative abundances of *Terrisporobacter*, and was negatively correlated with the relative abundance of *Buchnera* and *Enterobacter*. It was also observed that ZO-1 was positively correlated with the relative abundances of *Buchnera* and *Enterobacter*, and negative correlations were observed with the relative abundance of *Romboutsia*, *Terrisporobacter* and *Veillonella*. Occludin was positively correlated with the relative abundances of *Buchnera*. Claudin-1 was positively correlated with the relative abundances of *Buchnera* and *Enterobacter*, although it was negatively correlated with the relative abundance of *Romboutsia* and *Terrisporobacter*. The ADG was positively correlated with the relative abundances of TLR4, ZO-1, and Claudin-1, and negative correlations were observed with the relative abundance of 3-Methoxy-4-Hydroxyphenylglycol Sulfate, L-Arginine, N-Acetylneuraminic acid, Pro-Ala, Uridine, IL-8, TNFα, and *Veillonella*.

## Discussion

### Effect of ES on weight gain, diarrhea incidence, jejunum inflammatory cytokines and barrier proteins of Bamei suckling piglets

This study demonstrates that feeding supplement milk replacer not only increased ADG of Bamei piglets, but also reduce the weaning days and diarrhea incidence rate, which is consistent with previous results [22]. The intestine serves as the first line of defense against toxins and antigens from ingested food. In the intestine, a multilayered barrier system (superficial mucus layer, an epithelial monolayer, the adherens-junctional complex system, the immune system, and gut microflora) are employed to prevent pathogens from invading the body [23]. Tight junction proteins, such as Occludin and Claudin-1, form a physical barrier by decreasing intestinal tract permeability [24]. Peng et al. found that the expression of tight junction protein coding genes (ZO-1, occludin and claudin-1) were increased by dietary intervention [25]. In the present study, early dietary supplementation significantly increased mRNA levels of the innate immune system pattern recognition receptor (TRL4) and barrier proteins (ZO-1, Occludin and Claudin-1) in suckling pigs by 21 days of age. This response could be speculated to be due to an increase in the proliferation and differentiation of jejunal epithelial cells. Early food introduction (i.e. milk replacer) would increase microbial fermentation, inhibit pathogen proliferation, and decrease colon inflammation risk [22]. This would stimulate gastrointestinal tract development by promoting adaptation to a complex dietary environment [26, 27]. In the present study, however, early food introduction decreased the expression of TNFα and IL-8. The decreased TNF-α and IL-8 expression might be due to increased jejunal metabolites. For example, when a pathogen invades, increased butyrate concentrations could stimulate macrophages to produce the anti-inflammatory cytokine IL-10 and simultaneously suppress the expression of the pro-inflammatory cytokines TNF-α and IL-6 [28]. Milk replacer supplementation increased the TLR4vexpression, which corroborates the observations of Shi et al. [22]. It has been hypothesized that early food introduction increases intestinal permeability and reduce ZO-1 expression [29]. However, early food supplementation did not enhance ZO-1, Occludin, and Claudin-1 expression. Therefore, the increased metabolite concentrations and altered microbial composition due to early food supplementation might affect intestinal health by modulating gene expression related to the barrier and innate immune function.

### Effect of ES on jejunum microbiome and Metabolome of Bamei suckling piglets

Previous studies have shown that pig gastrointestinal tract microbial distribution varies among the intestinal segments, as well as, between the lumen and mucosa [30]. In monogastrics, the number of stomach and duodenum bacteria ranged from 10^1^ to 10^3^ CFU/mL, whereas jejunum and ileum concentrations were approximately 10^4^–10^7^ CFU/mL. Compared with the number of microorganisms in the stomach and small intestines, the numbers of bacteria in the colon (estimated to be 10^11^–10^12^ CFU/mL) are relatively high [31]. The jejunum is the main site of digestion and nutrition absorption. Compared with colon, the study of microbial diversity is less. In addition, the jejunum development in suckling piglets directly affects the growth performance after weaning. Therefore, the composition and distribution of the jejunal microflora as well as the spatial changes of the intestinal microflora in suckling piglets is very important and needs further study. In this study, responses to early dietary supplementation on the gut microbiome and its metabolic profiles were studied by using high-throughput 16S rRNA gene sequencing and LC-MS analysis. The data clearly demonstrate that early supplementation resulted in a significant increase in gut microbial diversity for the suckling Bamei piglets. In addition, the improved gut bacteria were highly correlated with changes of several gut microflora-related metabolites, indicating that early dietary supplementation not only improves gut bacteria at the abundance level, but also substantially alters the metabolomic profile of the gut microbiome. These intestinal alterations result in improved homeostasis of host metabolites of the newborn animal by early supplementary feeding. These findings provide mechanistic insights for improving the early intestinal microbiota of newborn animals.

Many studies have shown that metabolite changes, gut microbiome interference, and the links between them are major risk factors leading to abnormal tissue functions and diseases, such as cancer [32], metabolic diseases [33], cardiovascular disease [34], insulin resistance [35], and obesity [36]. The gut microbiome apparently can directly alter its metabolic capacity to impact intestinal functions locally through microbial products. For example, changes in the gut microbiota increased deoxycholic acid concentrations. The enterohepatic circulation of deoxycholic acid causes a senescence-associated secretory phenotype in hepatic stellate cells, which promotes the development of hepatocellular carcinoma in mice exposed to chemical carcinogens [32]. Likewise, altered gut microbiota have appeared to trigger systemic effects on host metabolism in remote tissues such as liver, brain, adipose, and muscle. This may be the result of microbial metabolic products either functioning as signaling molecules or acting in conjunction with the host metabolism of diverse chemicals affecting disease susceptibility. For instance, leukemia has been reported to increase the production of the adipokine IGFBP1, reduce incretin activity, short chain fatty acids, and 5-hydroxytryptamine production, causes intestinal flora imbalances to promote insulin resistance, the systemic destruction of glucose metabolism, and hijacking host glucose in order to promote tumor growth [37].

Newborn piglets represent a critical stage in swine production, which is characterized by rapid metabolism and growth. Unfortunately, the sow’s milk production is generally unable to meet the nutritional demands to meet the nutrient requirements of each piglet for optimum growth. Therefore, providing piglets with nutritional supplementation during lactation and nursing is essential to provide the nutrient to support the rapid growth [38]. Unfortunately, the developing structure and function of piglets’ gastrointestinal tract are not fully developed during this life stage to maximize nutrient absorption. Changes to the external environment, including management stresses (weaning from sows, group transfers, etc.), changing diets (from liquid mother’s milk to solid feed), disease stresses causing diarrhea, and immune challenges may all be manifest as diseases. Studies have shown that feeding milk replacer (ES) can increase feed intake, weaning weight and uniformity, reduce diarrhea and mortality, and promote piglet growth [39, 40]. The present study demonstrated similar results. In addition, a decreased diarrhea incidence was observed for ES fed piglets. These results demonstrate the potential feeding a milk replacer supplement (ES) to promote the growth performance of Bamei suckling piglets.

The intestine contains a large number of microorganisms, which play an important role in human and animal health. Host genetics and external factors such as delivery mode [41, 42], environment [9], nursing milk [43], and solid feed [13] have been reported to contribute to the development of the gut microbiota of infants. Compared with adult intestinal microflora, the neonatal intestinal microflora is truly a dynamic state. However, the neonatal intestinal microflora an important role in the development of intestinal functioning and the innate immune system. It not only poses a benefit and a risk to neonates, but may also have short- and long-term effects on health in later life [44, 45]. In this study, it was observed that feeding a milk replacer supplement (ES) substantially improved intestinal microbiota, however the results indicated no differences in diversity indices of the jejunal microbiota between the two groups. Although, for piglets fed ES the richness index was increased. These observations are in contrast to those reported in a previous study in which the diversity (Shannon) and richness (Chaol) indices of gut microbiota in Hu lambs with alfalfa intervention was similar to control [16]. The differences between the two studies might be due to the different animal models studied, i.e. nonruminant versus ruminant. Consistent with previous research [46], exclusively breastfed infants had lower total bacterial counts than those receiving other nutritional forms. In the present study, the difference in microbial composition between the two treatment groups on day 21 was due to the differences in feeding programs. Some studies have found that the milk replacer supplementation significantly decreased the relative abundance of *Lactobacillus* [22]. In the present study, *Romboutsia*, *Actinobacillus*, and *Lactobacillus* were reduced for piglets fed ES. The differences in *Romboutsia, Bacteroides* and *Lactobacillus* abundances may be the result of the different forages used in those studies [13, 47]. The decreased *Lactobacillus* abundance may be the result of decreased oligosaccharide ingestion, which has been associated with a reduction in breast milk consumption after early food introduction [48]. These results indicate that Bamei piglets consuming ES altered the gut microbiota by altering the beneficial bacterial colony structure [13]. Therefore, it is reasonable to hypothesize that the differences in gut microflora are the result of early dietary intervention, host–microbe interactions, and/or the host’s physiological state. Furthermore, it is possible that the most simportant host–microbe interactions occur at the gut barrier.

Besides affecting the composition of jejunal microbiota, ES also altered the metabolism of jejunal microbiota. In this study, microbiota functional predictions indicated that the jejunal microbiota for piglets fed ES had higher enrichments of metabolic pathways involved in lipid metabolism, energy metabolism, and terpenoid and polyketide metabolism. Specifically, enriched arginine biosynthesis, pyrimidine metabolism, primary bile acid biosynthesis, valine, leucine and isoleucine biosynthesis, alanine, aspartate and glutamate metabolism, linoleic acid metabolism, taurine and hypotaurine metabolism, pantothenate and CoA biosynthesis, and riboflavin metabolism for piglets fed ES.

### Pearson’s correlation between jejunal contents bacterial communities and metabolites of Bamei suckling piglets

The Pearson’s correlation analysis suggested that the changes in jejunal microbial abundance induced by piglets fed ES resulted in a shift in the microbial metabolome. Nucleotide metabolites were significantly altered in jejunal contents after early supplementary for ES fed piglets. The presence of the altered nucleotide metabolites was highly correlated with changes in the gut bacterial genera. For example, uridine is a gastro-intestinal metabolite in mammals, a large part of which is derived from the degradation of RNA, and then further metabolized to uridine monophosphate [49]. Dietary supplementation of uridine could improve the intestinal barrier integrity and temper the degree of intestinal apoptosis. This could have the desired effects of reducing diarrhea incidence and improving weaned pig growth performance [50]. In the present study, uridine levels were positively correlated with *Bacteroides* and *Tyzzerella*. Decreases in uridine concentrations may be related to the change of dietary composition in the suckling piglets. Lipid, amino acid and carbohydrate metabolites are also sensitive to changes in the gut microbiome.

In this study, feeding piglets an early supplementary milk replacer has been shown to alter the gut microbiome and related metabolomic profiles. However, further study is necessary to elucidate the specific mechanisms. As demonstrated using correlation analysis between intestinal bacteria and metabolites, early supplementary piglet feeding can induce changes in gut microbiome abundance, leading to alterations in metabolic nutrients. Of equal importance, early supplementary piglet feeding may result in altered metabolome by altering gut bacteria physiology without altering species or relative abundance. Therefore, changes in metabolic profiles of the gut microbiome may not entirely depend on shifts in the microbial spectrum, as was revealed by 16S rRNA sequencing. This metabolic change via early supplementary feeding improving the gut microbiome and its function could be the result of altering other mechanisms, such as: gut bacteria types and numbers, gene regulation and protein expression. In HMP2, the microbial and host metabolites, proteomes, transcriptomes, epigenomes and serological characteristics of 132 human fecal samples, intestinal biopsies, and blood samples were analyzed to reveal the flora-host dynamic interactions [51]. Therefore, a multi-omics joint analysis may be necessary to clarify the role of early feeding supplementation for improving the intestinal microbiome function of Huzhu Bamei piglets.

The study results clearly demonstrate that early supplementary feeding altered the gut microbiome composition and related metabolic profiles in piglets. This is the first and critical step in understanding how early supplementary feeding affects the gut microbiome and its functions. Future studies are needed to address mechanisms and whether gut microbiome changes and associated metabolites occur in weaned piglets that received early supplementary food is the result of gut microbiome differences between suckling and weaned piglets. In this experiment, suckling Bamei piglets were provided a supplemental milk replacer on the 7th day of life. However, other studies have demonstrated that feeding time also affected intestinal microbiota [52]. Thus, changes in the intestinal microbiota may be related to feeding time. Likewise, the impact of additional factors should be the focus of future research.

## Conclusions

In conclusion, feeding Bamei suckling piglets a supplementary milk replacer improved ADG in combination with altering jejunum inflammatory cytokines, barrier proteins, and metabolomics and microbiome associations between specific bacterial genera and metabolites. Integrative information about the interactions between certain metabolites and microbial composition in the piglet jejunum could provide a better understanding of jejunal metabolites and microbial functions that contribute to the development of new management strategies for feeding Bamei suckling piglets. Furthermore, understanding the causes and mechanisms driving the interactions among jejunal bacteria and jejunum metabolism merits further investigation.

## Methods

### Animals and experimental design

A total of 10 purebred Bamei sows (healthy, 3–4 years old, 5-6th parity) were selected from a national seed breeding farm of Bamei pigs (Qinghai, China), which were housed individually, and the farrowing house environment was kept warm, ventilated, and regularly disinfected and cleaned. The sows were randomly assigned to one of two groups, and were provided identical diets (Table S2). In the control group (Con), suckling piglets did not receive dietary supplementation (birth weight = 0.88 ± 0.13 kg). Piglets in the early supplementary feeding group (ES) received a milk replacer (Beijing Dabeinong Science and Technology Group Co., Ltd., China) on the 7th day of birth, ad libitum (birth weight = 0.89 ± 0.12 kg). A small unit restriction bar was used to supplement the Baimei piglets, to ensure that each piglet has its own diet bar, and each piglet was fed with the same milk substitute, 6 times each day for 10 min. The milk replacer nutritional composition is given in Table S3. The piglets were housed with their sows prior to weaning. Piglets were weighed at the beginning and the end of the experiment to determine the average daily gain (ADG). The diarrhea incidence (%) as calculated as follows: (number of piglets with diarrhea × number of days with diarrhea)/ (number of piglets in the pen × 14 days) × 100% [53].

### Sample collection

At 21 days, the number of piglets, ES = 54, and Con = 58. 18 piglets with a similar weight of 5.01 kg in ES group and 17 piglets with a similar weight of 4.66 kg in Con group were euthanized with sodium pentobarbital (50 mg/kg) after a fasting period of 12-h on day 21 of lactation (Con = 17, ES = 18). The jejunal contents were collected, placed into 1.5 mL sterile polypropylene tubes, stored in liquid nitrogen for further analysis of the microbiome and metabolome. The corpse was placed in a special plastic bag, knotted, sealed, and stored at − 20 °C at the Experimental Animal Center of Gansu Agricultural University. Finally, corpses were transported to a professional sanitation plant for harmless incineration (Lanzhou China). Unskilled piglets were raised to 6 months of age and harvested for commercial use.

### 16S rRNA gene sequencing and KEGG analysis

The specific steps were reported in previous research studies [54], total bacteria DNA was extracted from the jejunum content samples using the PowerSoil® DNA Isolation Kit (MO BIO Laboratories, Inc., Carlsbad, CA, USA) according to the manufacturer’s instruction, and was stored at − 80 °C until the time of analysis. Sequencing of the16S rRNA gene was outsourced to BIOMARKER (Beijing, China). Illumina HiSeq 2500 sequencing of 16S rRNA gene was used to characterize microbial diversity and community composition. Using the extracted DNA as a template, PCR was performed using barcode primers located on both sides of the V_3_-V_4_ hypervariable region of the bacterial 16S rRNA gene. The primer sequences used are as follows: 338F: 5′-ACTCCTACGGGAGGCAGCA-3′ and 806R: 5′-GGACTACHVGGGTWTCTAAT-3′. Amplification was performed for 30 cycles using a DNA thermal Cycler (Bio-Rad, USA). The first cycle was at 98 °C for 2 min followed by 30 subsequent cycles of 98 °C × 30 s, 50 °C × 30 s, then 72 °C × 1 min, and the last cycle at 72 °C for 7 min.

The raw sequencing reads from the original DNA fragments were merged using FLASH v1.2.7, and assigned to each sample according to the unique barcodes. High-quality reads were selected and used for bioinformatics analysis. Each unique read from each sample was clustered into operational taxonomic units (OTUs) based on a 97% sequence similarity, as determined by UCHIME v4.2. For alpha diversity analysis, the OTU was rarified based on several metrics, including curves of OTU rank, rarefaction and Shannon, and calculated indices of Shannon, Chao1, Simpson, and ACE. For beta diversity analysis, the Non-Metric Multi-Dimensional Scaling (NMDS, Stress< 0.2, it shows that NMDS analysis has certain reliability [55]) and unweighted pair group method with arithmetic mean (UPGMA) were performed using QIIME based weighted uniFrac distance. Line Discriminant Analysis (LDA) Effect Size (LEfSe) was used to search for biomarkers that exhibited statistical differences. A Wilcoxon rank-sum test was used to determine significance, FDR corrects *p*-value.

Functional annotation and classification of all identified microbiomes were determined using pathway analyses, which was extracted using the search pathway tool in the KEGG Mapper platform (http://www.genome.jp/kegg/mapper.html). The pathway enrichment statistics were calculated using Fisher’s exact test, and the pathways with a corrected *p* value < 0.05 were considered to be the most significant pathways.

### LC-MS/MS metabolomics analysis

Metabolic profiling of samples was performed on an Agilent 1290 Infinity LC system (Agilent Technologies, Santa-Clara, California, USA) coupled with an AB SCIEX Triple TOF 6600 System (AB SCIEX, Framingham, MA, USA) in Shanghai Applied Protein Technology Co., Ltd.

### Preparation of samples for LC-MS analysis

The jejunal content samples (60 mg) were thawed at 4 °C, 200 μL ultrapure water was added to aid in homogenization of each sample. Then, 800 μL of methanol/acetonitrile (1:1, v/v) was added, and the samples were vortexed, and then sonicated on ice. The samples were then incubated at − 20 °C for 1 h to remove the protein, and then centrifuged for 15 min (13,000 x g, 4 °C), The supernatants were dried in a vacuum and stored at − 80 °C. The quality control (QC) samples were prepared similarly. For the UHPLC-Q-TOF/MS analysis, the samples were re-dissolved in 100 μL acetonitrile/water (1:1, v/v) solvent. To monitor the stability and repeatability of instrument analysis, QC samples were prepared by pooling 10 μL of each sample and these were analyzed together with the other samples. The QC samples were inserted regularly and analyzed per every ten samples.

### LC-MS/MS analysis

Metabolomic analysis process has been reported [56, 57]. For HILIC separation, samples were analyzed using a 2.1 mm × 100 mm ACQUIY UPLC BEH 1.7 μm column (waters, Ireland). In both ESI positive and negative modes, the mobile phase contained A = 25 mM ammonium acetate and 25 mM ammonium hydroxide in water and B = acetonitrile. The gradient was 85% B for 1 min, and was then linearly reduced to 65% by 11 min, and then further reduced to 40% in 0.1 min. This concentration was maintained for 4 min, and then increased to 85% in 0.1 min, with a 5 min re-equilibration period.

The ESI source conditions were set as follows: Ion Source Gas1 (Gas1) = 60, Ion Source Gas2 (Gas2) = 60, curtain gas (CUR) = 30, source temperature = 600 °C, IonSpray Voltage Floating (ISVF) ± 5500 V. In MS only acquisition, the instrument was set to acquire over the m/z range of 60–1000 Da, and the accumulation time for the TOF MS scan was set at 0.20 s/spectra. In auto MS/MS acquisition, the instrument was set to acquire over the m/z range 25–1000 Da, and the accumulation time for product ion scan was set at 0.05 s/spectra. The product ion scan was acquired using information dependent acquisition (IDA), with the high sensitivity mode selected. The parameters were set as follows: the collision energy (CE) was fixed at 35 V with ±15 eV; declustering potential (DP) was 60 V (+) and − 60 V (−); exclude isotopes within 4 Da, candidate ions to monitor per cycle: 10.

The initial UPLC-Q-TOF/MS data were converted into. mzXML format by ProteoWizard MSConvert processed using XCMS for feature detection, retention time correction and alignment. Minfrac was set as 0.5. The metabolites were identified by accuracy mass and MS/MS data which were matched with the lab database. After being normalized and integrated by using SVR and Peratoscaling method, the processed data were imported into SIMCA-P 14.1 (Umetrics, Umea, Sweden) for multivariate statistical analyses including principal component analysis (PCA) and partial least squares discriminant analysis (PLS-DA) as well as orthogonal partial least squares discriminant analysis (OPLS-DA). The significant different metabolites were determined based on the combination of a statistically significant threshold of variable influence on projection (VIP) values obtained from OPLS-DA model and two-tailed Student’s t-test (pvalue) on the raw data, and the metabolites with VIP values larger than 1.0 and pvalues less than 0.1 were considered as significant. Metabolic pathway enrichment analysis was performed using the OmicShare tools, a free online platform for data analysis (http://www.omicshare.com/tools).

### Quantitative real-time PCR

Total RNA was extracted from jejunal tissue using the Trizol reagent (TransGen). reverse-transcription was performed using the Transcript First-Strand cDNA Synthesis SuperMix Kit (TransGen). Expression of mRNA was quantified by real-time PCR. Primers targeting 6 genes were designed using the Oligo 7.0 program (Table S4). The primers were synthesized by the Suzhou Jinweizhi Biotechnology Co., Ltd. The SYBR® Premix Ex TaqTM was purchased from Takara Biomedical Technology (Beijing) Co., Ltd. Real-time PCR was performed in a LightCycler 480 (Roche Diagnostics, Mannheim, Germany) Real-time System. The amplification of β-actin was used as an endogenous control gene for each sample to normalize the expression of the selected genes. The 2^-ΔΔCT^ method was used to analyze the data. The PCR conditions were: one cycle at 95 °C for 30 s, 40 cycles at 95 °C for 5 s, 60 °C for 20 s, and 72 °C for 15 s, and one cycle at 65 °C for 15 s. The PCR reactions total volume were 20.00 μL: SYBR® Premix Ex Taq™ 10.00 μL, cDNA 2.00 μL, Forward Primer (10 μmol/L) 0.80 μL, Reverse Primer (10 μmol/L) 0.80 μL, ddH_2_O 6.40 μL.

### Statistical analyses

The results of weight gain, incidence of diarrhea, and mRNA expression related to the jejunal barrier were presented as means ± SD. The statistical analysis was performed using SPSS, version 21. Differences between two groups were analyzed using Student’s t-test. Results were considered statistically significant when *p* < 0.05. The correlation matrix between the jejunum bacterial species and ADG, mRNA expression levels of inflammatory cytokines and barrier proteins, jejunum microflora-related metabolites was generated using Pearson’s correlation coefficient.

## Supplementary information

**Additional file 1: Table S1.** Differences in metabolites in the jejunum of Bamei suckling piglets without feeding or early supplementary feeding.

**Additional file 2: Table S2.** Composition and nutrient levels of diets^a^

**Additional file 3: Table S3.** The nutritional values of the commercial milk replacer

**Additional file 4: Table S4.** Sequences of forward and reverse primers used for real-time PCR.

## Data Availability

Metabolome raw sequence data were uploaded to the MetaboLights database and are available through accession number MTBLS1698.Microbiome raw sequence data were uploaded to the National Center for Biotechnology Information (NCBI) database and are available through accession number SRS5958740.

## Abbreviations

PCA: Principal Component Analysis
LDA: Least discriminant analysis
ADG: Average daily gain
OTUs: Operational Taxonomic Units
KEGG: Kyoto Encyclopedia of Genes and Genomes
NMDS: Non-MetricMulti-Dimensional Scaling
LC-MS: Liquid chromatograph-mass spectrometry
PLS-DA: Partial Least Squares Discrimination Analysis
UPGMA: Unweighted Pair-group Method with Arithmetic Mean
16S rRNA: 16S ribosomal ribonucleic acid
